# High prevalence of right to left shunt in women with chronic migraine

**DOI:** 10.1186/1129-2377-14-S1-P105

**Published:** 2013-02-21

**Authors:** D Larrosa, L Benavente, S Calleja, M Gonzalez-Delgado, J Pascual

**Affiliations:** 1 University Hospital Central de Asturias, Spain

## Introduction

Prevalence of right to left shunt is estimated as 25% of the general population [1]. The prevalence of shunt has been shown to be increased in migraine with aura and one study has found a high prevalence of shunt in chronic migraine (CM) [2].

## Objectives

To study the prevalence of right to left shunt in a series of women with CM.

## Methods

This series includes 51 women (age 44 years, range 16-63) meeting diagnostic criteria (IHC-II revised 2006)for CM. There were only 5 women with migraine with aura attacks. We carried out a transcranial doppler study (AplioXG, model SSA-790A, Toshiba) following the CODICIA study protocol [3].

## Results

Thirty patients (58.8%) showed some degree of right to left shunt. Three (60%) out of the 5 patients with migraine with aura had shunt. Thirteen patients (43%, 25 of total series) had hits during normal breathing and 17 (58%, 33% of the total series) during Valsalva maneuver. Shunt was massive in 10 patients (33%, 20% of the total series); in 9 of them shunt became massive only during the Valsalva maneuver.

## Conclusions

Prevalence of right to left shunt in women with CM is higher than expected for the general population (1). The clinical implications of our findings need to be determined, though they suggest a relationship between the presence of shunt with an increased frequency of migraine attacks.
